# Pulmonary, circulatory and renal considerations in the early postoperative management of the lung transplant recipient

**DOI:** 10.21542/gcsp.2023.18

**Published:** 2023-08-01

**Authors:** Reda E. Girgis, Ryan J. Hadley, Edward T. Murphy

**Affiliations:** Richard DeVos Lung Transplant Program, Corewell Health West, Michigan State University, College of Human Medicine, Grand Rapids, Michigan, USA

## Abstract

Lung transplantation volumes and survival rates continue to increase worldwide. Primary graft dysfunction (PGD) and acute kidney injury (AKI) are common early postoperative complications that significantly affect short-term mortality and long-term outcomes. These conditions share overlapping risk factors and are driven, in part, by circulatory derangements. The prevalence of severe PGD is up to 20% and is the leading cause of early death. Patients with pulmonary hypertension are at a higher risk. Prevention and management are based on principles learned from acute lung injury of other causes. Targeting the lowest effective cardiac filling pressure will reduce alveolar edema formation in the setting of increased pulmonary capillary permeability. AKI is reported in up to one-half of lung transplant recipients and is strongly associated with one-year mortality as well as long-term chronic kidney disease. Optimization of renal perfusion is critical to reduce the incidence and severity of AKI. In this review, we highlight key early post-transplant pulmonary, circulatory, and renal perturbations and our center’s management approach.

## Introduction

Lung transplantation (LTx) has witnessed remarkable growth over the last two decades. In the U.S., the annual volume has grown from 1,172 in 2004 to over 2,700 in 2019^1^, largely due to more efficient utilization and allocation of donor grafts in addition to the expansion of recipient criteria. Concomitant with the increased numbers of patients, survival has also improved. The most recent one-year mortality nationally in the U.S. was 11%^2^, compared with 23% in 2000^3^, reflecting refinements in organ procurement, transplant surgical techniques, and peri-operative care.

**Figure 1.**
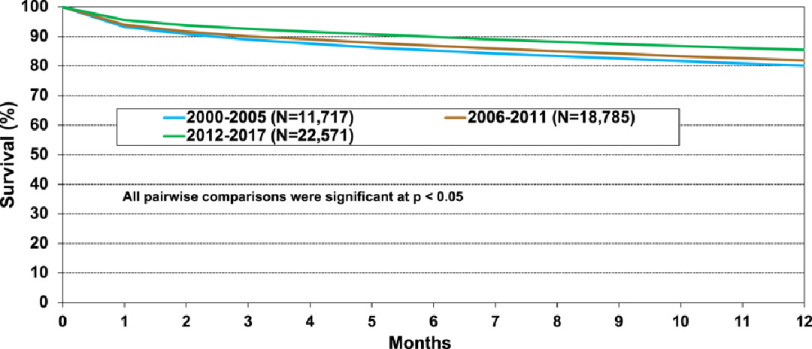
Kaplan–Meier survival within 12 months of adult lung transplant by era based on the International Society of Heart and Lung Transplant Registry. While early survival has improved over time, the risk of death remains high during the early postoperative period. Most subsequent deaths in the first year are often related to severe early post-transplant complications. Reproduced from https://ishltregistries.org/registries/slides.asp, 2021.

Nevertheless, the risk of death remains highest during the early postoperative period (Figure 1), predominantly related to graft failure, cardiovascular causes, multi-organ failure, and infection^4,5^. Primary graft dysfunction (PGD) and acute kidney injury (AKI) are important complications with overlapping risk factors that often set the stage for prolonged intensive care unit stay, additional insults, infections, iatrogenic complications, and ultimately, mortality. Sequelae extend well beyond the initial postoperative period. Recognition of the pathophysiological mechanisms involved allows targeted interventions to reduce the incidence and severity of both conditions. Strategies to closely monitor and optimize multiple complex cardio-pulmonary-renal interactions^6^ during this critical phase^7^ offers an important opportunity to improve long-term outcomes. In this article, we review key early post-transplant pulmonary, circulatory, and renal perturbations, and our center’s management approach (Table 1).

**Table 1:** Principles of early post-operative management.

**Respiratory**
• Minimize FiO_2_
• Protective mechanical ventilation (using donor PBW for tidal volume)
• Avoid overventilation and ensure adequate expiratory time (for COPD single lung transplant)
• Early spontaneous awakening trial
• Adequate analgesia
• Rapid liberation from mechanical ventilation if appropriate
• Extubation to high-flow nasal cannula and/or non-invasive ventilation in high-risk subjects
• Aggressive airway clearance techniques
• Early mobilization and minimization of sedation
• Grade PGD at time 0, 24, 48 and 72 hrs
• Conservative fluid management strategy
• Early institution of ECMO support for refractory PGD
**Circulatory**
• Close invasive hemodynamic monitoring and assessment of tissue perfusion
• Ensure adequate, but not excessive pre-load
• Nor-epinephrine 1^st^ line vasopressor. Supplement with vasopressin
• Low threshold for angiotensin II
• Prevention and early control of atrial arrythmia
**Renal**
• Close monitoring of serum creatinine and urine output
• Optimization of volume status and renal perfusion
• Avoid nephrotoxic agents, including radiocontrast dye
• Glycemic control

## Pulmonary considerations

### Ventilator management

Ventilatory management of the post-lung transplant recipient begins in the operating room. Upon graft reperfusion, the lowest FiO_2_ required for adequate oxygenation should be used to reduce the risk of primary graft dysfunction^8^. Minimizing oxygen use throughout the postoperative course reduces the risk of resorption atelectasis and oxidative injury. In a large observational study of critically ill patients, hyperoxemia (arterial oxygen tension > 100 mmHg) was strongly associated with mortality, irrespective of underlying respiratory diagnosis or mechanical ventilation^9^.

While no adequately controlled studies have been performed in the lung transplant setting, a protective lung ventilation strategy with low tidal volumes should be applied both intra- and postoperatively, given the benefits observed in other populations at risk of acute lung injury and other pulmonary complications^10^. An initial tidal volume of 6–8 ml/kg predicted body weight (PBW) *of the donor* is suggested for bilateral recipients and 4–6 ml/kg for single-lung recipients. Using recipient PBW risks the application of potentially injurious tidal volumes when the donor is undersized^11^. We prefer the volume-cycled assist-control mode over the pressure-cycled mode to avoid large variations in tidal volume with changing respiratory mechanics. The initial positive end-expiratory pressure (PEEP) is typically 5–10 cm H_2_O, with subsequent adjustments based on the FiO_2_ requirement following the ARDSnet algorithm^12^. Plateau pressures should be maintained at <30 cm H_2_O by limiting tidal volume and/or improving respiratory system compliance, if required. A limit on peak inspiratory pressure to <35 cm H_2_O is reported by most programs^11^. While prolonged mechanical ventilation after LTx has been associated with airway complications, there are no data regarding the impact of ventilating pressures^13,14^.

There is increasing evidence that driving pressure (plateau –  PEEP or ΔP) may be the most important factor in attenuating lung injury in adult respiratory distress syndrome (ARDS), independent of tidal volume, plateau pressure, or PEEP^15^. Similarly, a meta-analysis of randomized controlled trials of different protective ventilation strategies during surgery found that the ΔP was the only ventilatory parameter associated with postoperative pulmonary complications^16^. Whether a strategy targeting a certain ΔP leads to improved outcomes in ARDS is currently being investigated. Our practice is to aim for ΔP < 15 cm H_2_O^17^.

Respiratory rate is set to achieve an adequate minute volume. In the absence of severe respiratory acidosis, the backup rate is kept 2–3 breaths below the patient’s spontaneous rate to allow setting of their own PaCO_2_. This is particularly important in pre-existing hypercapnia, where rapid normalization of PaCO_2_ results in alkalemia. As renal compensation occurs within 12–24 h, subsequent ventilator weaning is often associated with respiratory acidosis, as the medullary respiratory center requires more time to adjust.

Native lung hyperinflation is an uncommon but potentially serious complication of single-lung transplantation for emphysema resulting from incomplete emptying prior to the onset of the next inspiration^18^. Ensuring a sufficiently low inspiratory/expiratory (I:E) time ratio is essential to prevent progressive hyperinflation and the potential for tamponade physiology and encroachment on the allograft. A lower respiratory rate prolongs the expiratory time and has the greatest effect on the I:E ratio. Lower tidal volume and faster inspiratory flow rates shorten inspiratory time, but have a smaller impact on reducing I:E. If severe, independent lung ventilation may be required. Early extubation is the best strategy for avoiding this problem^18^.

Following an uncomplicated transplantation procedure, rapid liberation from mechanical ventilation should be pursued. Sedation is stopped if tolerated. Immediate interruption of sedation in critically ill patients after abdominal surgery resulted in a marked reduction in the time to successful extubation compared with usual care (median, 8 vs. 50 h)^19^. The use of opiate analgesics is minimized with intercostal nerve cryoablation during surgery or early epidural catheter placement for inadequate pain control^20,21^. If PaO_2_/FiO_2_ ratio exceeds 200, the chest radiograph has no major abnormalizes, and hemodynamics are stable, then ventilator modality is changed to pressure-support adjusted to an adequate tidal volume and respiratory rate^22^. Extubation can proceed if mental status is normal and there is good cough with minimal secretions after a 30-minute spontaneous breathing trial^23^. Some programs have instituted protocols for immediate extubation in the operating room for selected patients^24,25^. When risk factors for re-intubation are present, extubation to non-invasive ventilation (NIV) is advised^23^. The use of a high-flow nasal cannula post-extubation is comparable to NIV in reducing failure^26–28^, while adverse effects such as gastric distention and nasal bridge ulceration are avoided. If invasive mechanical ventilation is required beyond 24 h, efforts to minimize sedation and encourage early mobilization are essential for reducing the duration of ventilation and resultant complications^23^. Early tracheostomy may facilitate these objectives and has been associated with earlier liberation and shorter ICU length of stay compared with late tracheostomy^29,30^.

The effects of lung denervation, disruption of the bronchial and lymphatic circulations and the impact of immunosuppressive drugs on mucociliary clearance^31^ makes pulmonary toilet maneuvers even more critical than in other postoperative patients. Variable degrees of airway ischemia in the watershed zone just distal to the anastomosis from devascularization, compounded by post-reperfusion hypotension, is common, resulting in epithelial sloughing, tenacious, adherent secretions, and mucous plugging. Incentive spirometry, positive expiratory pressure devices, and active cycle of breathing techniques^32^ should be routinely employed immediately after extubation, ideally with the patient sitting upright and leaning forward with feet supporting weight to facilitate ventilation of the lung bases. Nebulized *β*_2_ agonists and hypertonic saline may facilitate the clearance of secretion. Early and progressive mobilization is critical. A low threshold for therapeutic bronchoscopy is maintained and can often be accomplished with minimal sedation. Attention to airway clearance techniques in intubated or tracheotomized patients is also important^32^.

**Figure 2.**
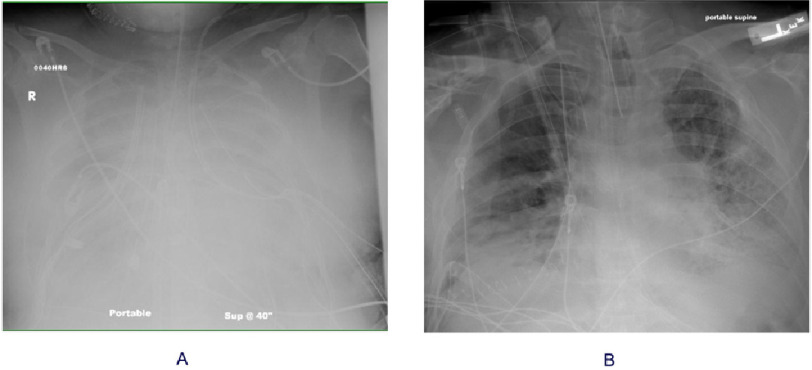
Representative chest radiographs of primary graft dysfunction. **Panel A**: Fulminant pulmonary edema 48 h after bilateral lung transplant for severe idiopathic pulmonary arterial hypertension and right heart failure. PaO_2_:FiO_2_ ratio < 100. Procedure performed with cardiopulmonary bypass and required large volume blood transfusion. Died on postoperative day 7. **Panel B**: Patchy right lung opacities consistent with pulmonary edema 72 h after right single lung transplant for idiopathic pulmonary fibrosis. PaO_2_:FiO_2_ ratio: 184. Procedure done without extra-corporeal support or blood transfusion. No pre-transplant pulmonary hypertension. Donor had active smoking and vaping history. Recipient duration of mechanical ventilation was 22 days. At 17 months follow-up, has stage I chronic lung allograft dysfunction, restrictive phenotype with pleural fibrosis.

#### Primary graft dysfunction

Primary graft dysfunction (PGD) is a form of non-cardiogenic pulmonary edema within the first 72 h after lung transplantation caused by pathological findings of acute lung injury with diffuse alveolar damage in the most severe cases^33^. Diagnosis is made based on the presence of radiographic opacities consistent with pulmonary edema (Figure 2) in the absence of other causes, such as left heart failure, pneumonia, pulmonary venous obstruction, hemorrhage, or hyperacute rejection^34^. Severity grading is based on the PaO_2_/FiO_2_ ratio analogous to that used for ARDS (Table 2). The incidence of all PGD grades is approximately 30% and 15–20% for grade 3 at 72 h. PGD represents the largest single cause of early deaths. The Lung Transplant Outcomes Group (LTOG), a large prospective multicenter registry, found that PGD grade 3 at 48 or 72 h was associated with a 90-d mortality of 23% vs. 5% among recipients with lower grades or no PGD^8^. Beyond its impact on early outcomes, PGD is an independent risk factor for subsequent chronic lung allograft dysfunction (CLAD)^35^, the main long-term cause of mortality.

**Table 2:** Classification schema for grading severity of primary graft dysfunction.

PGD grade	Pulmonary edema on chest radiograph	PaO_2_:FiO_2_ ratio	SpO_2_:FiO_2_ ratio^a^
0	No	>300	>315
1	Yes	>300	>315
2	Yes	200–300	235–315
3	Yes	<200	<235

**Notes.**

Abbreviations FiO_2_ Fraction of inspired oxygen PaO_2_ Partial pressure of arterial oxygen PGD Primary graft dysfunction SpO_2_ Peripheral capillary oxygen saturation

a If PaO_2_ measurement is not readily available.

ECLS with supportive radiographic findings as grade 3.

Several risk factors have been described with variable associations for the development of PGD that can be divided into donor-related, recipient-related^36^, peri-operative, and post-transplant (Table 3)^33^. LTOG analysis identified eight independent variables (Table 3)^8^. Recipient-related factors appear to be most important, however, donor variables play a larger role in higher risk recipients. The LTOG group developed a simple 3-variable model where recipients with a BMI < 25, a diagnosis of chronic obstructive lung disease or cystic fibrosis and mean pulmonary artery pressure < 40 mmHg had a PGD grade 3 incidence at 48-72 h of 4–7% whereas all others had a predicted incidence of 15–18%. Adding donor smoking to the model did not impact PGD risk in low-risk recipients, but significantly increased the incidence in high-risk recipients^37^. A single-center report demonstrated that lungs from donors aged ≥ 55 years were associated with higher early mortality (PGD was not specifically reported) among recipients with pulmonary hypertension (PH) or cardiopulmonary bypass (CPB) time > 4 h, yet no increased risk for older donors in the absence of PH or prolonged CPB^38^. Similarly, in an analysis of the United Network for Organ Sharing (UNOS) registry, the use of “extended criteria” donors (this term includes older donor age and other variables) was associated with greater 1-yr mortality, particularly among recipients with a very high lung allocation score, which reflects the severity of underlying disease^39^. These findings support the concept that low-risk donors are preferred for recipients at the greatest risk of severe PGD^40^.

**Table 3:** Risk Factors for PGD.

Category	Risk Factor
Donor related	Cigarette smoking history*
	Undersized donor relative to recipient (pTLC ratio <1)†
	Extremes of age (pediatric and age >55 y)
	African American race
	Heavy alcohol use
	Head trauma or aspiration as cause of death
	Chest trauma or lung contusion
	Smoke exposure
	Prolonged mechanical ventilation
	Hemodynamic instability after brain death
	Massive donor blood transfusion (>10 units)
	Low donor BMI (<18.5 kg/m^2^; ≥30 protective)
Recipient related	Underlying disease of PAH or sarcoid*
	Elevated pulmonary artery pressure*
	BMI >30 kg/m^2^*
	Left ventricular diastolic dysfunction†
	Underlying disease of IPF
	African American race
	Female sex
Intra-operative	Use of cardiopulmonary bypass*
	Single lung transplant*
	Packed RBC transfusion >1 L*
	Reperfusion FiO_2_ >0.4*
	Use of Euro-Collins preservations solution
	Prolonged ischemic time
Post-transplant factors that can exacerbate or mimic PGD	Volume overload and increased left atrial pressure
	Aspiration
	Pulmonary venous anastomotic complications
	Pneumonia
	Hypotension/shock
Injurious mechanical ventilation

It is evident that CPB is a strong risk factor for PGD. The mechanism(s) of acute lung injury after CPB for cardiac surgery involves a systemic inflammatory response related to contact with the extracorporeal circuit^41^. Recipients requiring CPB often have PH and are more likely to receive blood transfusions, further increasing the risk of PGD. In a multicenter registry report, the incidence of severe PGD following the use of veno-arterial extracorporeal membrane oxygenation (VA-ECMO) for intra-operative support was 29% compared to 43% after CPB and 12% off-pump^42^, consistent with other studies supporting the benefits of VA-ECMO over CPB^43^. We do not routinely use extracorporeal support if single-lung ventilation can be safely accomplished based on the response of pulmonary artery pressure and right ventricular size and function during continuous Swan-Ganz catheter and transesophageal echocardiographic (TEE) monitoring to pulmonary artery clamping. Inhaled nitric oxide can be useful in facilitating single-lung ventilation in borderline cases. When required, VA-ECMO often provides adequate cardiopulmonary support without the level of anticoagulation needed for CPB, thus also minimizing transfusion. Pulsatile blood flow is ensured after reperfusion of the graft. In our practice, CPB is only utilized when its large blood volume reservoir capacity is required, as in massive hemorrhage, the need for a bloodless field, for example, repair of proximal pulmonary artery tear, or concomitant complex cardiac procedures. Coronary artery bypass grafting can often be accomplished off-pump or using VA-ECMO.

Transplant indications of pulmonary arterial hypertension (PAH) and PH per se are strong risk factors for severe PGD^8,44^. The underlying mechanism appears, at least in part, due tocardiac remodelling. Longstanding severe PH leads to right ventricular (RV) dilatation and dysfunction in response to an elevated afterload, while intrinsic contractility is often greater than normal^45^. Chronic underfilling of the left ventricle (LV) leads to atrophy and impaired contractility^46,47^. After relief of RV afterload with lung transplantation, the LV may be unable to accommodate the sudden increase in pulmonary venous return, with frequent occurrence of transient LV dysfunction^48–51^. Even a small rise in left atrial pressure in the setting of increased capillary permeability would be expected to promote alveolar edema. Further supporting the role of left heart post-LTX for PAH is the finding of worse early outcomes in recipients with LV diastolic dysfunction pre-transplant^52^. Echocardiographically defined LV diastolic dysfunction pre-transplant was also associated with severe PGD in the entire LTOG cohort^53^.

**Table 4:** Simplified conservative fluid management protocol (fluid and catheter treatment trial lite).

		Mean arterial pressure ≥60 mm Hg and off vasopressors ≥12 Hr	
Central Venous Pressure (Recommended)	Pulmonary Artery Occlusion Pressure (optional)	Urine Output <0.5 ml/kg/hr	Urine Output ≥0.5 ml/kg/hr
>8	>12	Furosemide^a^; reassess in 1 hr	Furosemide^a^; reassess in 4 hr
4–8	8–12	Give fluid bolus; reassess in 1 hr	Furosemide^a^; reassess in 4 hr
<4	<8	Give fluid bolus; reassess in 1 hr	No interventions; reassess in 4 hr

**Notes.**

a Recommended furosemide dosing = begin with 20 mg bolus or 3 mg/hr infusion or last known effective dose. Double each subsequent dose until goal achieved (oliguria reversal or intravascular pressure target) or maximum infusion rate 24 mg/hr or 160 mg bolus reached. Do not exceed 620 mg/d. Also, if patient has heart failure, consider treatment with dobutamine.

A strategy of pre-emptive or prophylactic awake VA-ECMO and extubation during the early postoperative period was shown to result in comparable short- and long-term outcomes in recipients with severe PH (where early mortality is historically the highest) compared to other recipients^54,55^. Central cannulation is converted to peripheral for continuation of ECMO support in the intensive care unit. VA-ECMO partially bypasses the central circulation, thereby reducing allograft perfusion, allowing a gradual increase in left heart venous return over several days and attenuating pulmonary edema formation. Our approach is to carefully gauge RV size and function and LV filling with TEE and assess for any signs of pulmonary edema formation during VA-ECMO weaning in the operating room. If cardiac and allograft functions are adequate, decannulation is performed, and PGD preventative measures are instituted with close monitoring.

Management of established PGD is largely based on experience from ARDS of other causes. The protective lung ventilation strategies outlined above were continued immediately postoperatively. The goal of low tidal volume should be balanced by the need for heavy sedation. Driving pressure (DP) may be a crucial factor in the development of ventilator-induced lung injury. Once manifestations of ALI begin to improve, tidal volume can be liberalized while targeting DP < 13–15 cm H_2_O. DP can be measured using passive and spontaneous breathing during pressure-support ventilation^56^. Besides sufficient tidal volume, other strategies to avoid ventilator dyssynchrony and the need for deep sedation include optimizing inspiratory flow and breath triggering^57^.

A growing body of evidence has emerged in support of a conservative fluid management strategy in acute lung injury since the ARDS network demonstrated a shorter duration of mechanical ventilation, improved gas exchange, and fewer ICU days without a difference in shock or renal failure in the Fluid and Catheter Treatment Trial (FACTT)^58^. A simplified protocol that relies on central venous pressure (CVP) and urine output in patients with adequate systemic blood pressure and off vasopressors ≥ 12 h (FACTT Lite, Table 4) resulted in equivalent efficacy and safety as the FACTT conservative arm^59^. In a cohort of 118 lung transplant recipients, those with a CVP less than or equal to the mean of 7 cm H_2_O had significantly shorter duration of mechanical ventilation and ICU length of stay and reduced ICU mortality^60^. The low CVP group also had lower serum creatinine levels and inotrope requirements. Fluid restriction was comparable to standard fluid therapy in a large international randomized trial on septic shock^61^. This data provides reassurance that limiting volume status and targeting lower cardiac filling pressure, thereby reducing transudation of alveolar edema in ALI, does not have negative consequences of reduced pre-load. Hence, we target a CVP of 7 or lower in PGD in the absence of demonstrable hypovolemia. Pressors are used to support blood pressure once the pre-load is optimized.

Inhaled pulmonary vasodilators have not been shown to be useful in the prevention of PGD^62,63^, but may transiently improve oxygenation when FiO_2_ and PEEP requirements are excessive^64^. The short-term use of neuromuscular blockade may be required. Prone positioning has been successfully applied in a small series^65^ but may be challenging in the early postoperative period. Institution of venovenous ECMO for refractory respiratory failure results in acceptable outcomes^66^. We apply conventional ECMO criteria for ARDS^67^: PaO_2_/FiO_2_ ratio < 50 for >3 h or < 80 for >6 h despite optimal PEEP or hypercapnic acidosis (pH < 7.25 for >6 h despite optimal ventilator settings. Other considerations include persistently high plateau pressures (> 35 cmH_2_O) and FiO_2_ requirements (>0.6). Concomitant shock may require VA-ECMO. The early application of ECMO within 48 h of transplant is associated with a dramatic reduction in hospital mortality compared to later initiation^68^. In addition to supporting gas exchange, early ECMO use allows a considerable reduction in the intensity of ventilator support with attendant lung injury^69^.

## Hemodynamic management

Lung transplantation is a hemodynamically stressful surgical procedure. Prolonged duration of anesthesia accompanied by extensive cardiac manipulation and wide swings in volume and/or the use of extracorporeal support often results in variable degrees of circulatory compromise early postoperatively. Close monitoring with a pulmonary artery catheter, arterial line, and Foley catheter is useful for the first 24-48 hrs to guide management. CVP monitoring and noninvasive assessments often suffice thereafter until cardiopulmonary-renal function stabilizes. Hemodynamic and O_2_ delivery targets are presented in Table 5^22^.

**Table 5:** Hemodynamic and O2 delivery targets.

• MAP: 65–75
• Cardiac index: 2–2.5 initially
• CVP <8 mm Hg
• PAWP or LAP <11 mm Hg
• Urine output: >0.5 ml/kg/hr
• SaO2 ≥ 90%
• Hgb ≥7.0 gm/dl

Significant postoperative hemorrhage is uncommon when adequate intra-operative hemostasis is achieved. Extracorporeal support, pleural adhesions or thickening due to previous thoracic surgery or underlying lung disease, and prominent bronchial collaterals, as observed in bronchiectasis and pulmonary vascular disease, increase the risk of major bleeding^70–73^. Thin serosanguinous pleural drainage is expected when extensive mediastinal lymph node dissection is required and with volume overload. Re-exploration is required for grossly bloody drainage at a rate of 500 ml in one hour, 250 ml/h for 2 h, 150 ml/h for 4 h, or any bleeding associated with hemodynamic instability and retained hemothorax. We avoid the use of concentrated coagulation factors because of the risk of thromboembolism, including pulmonary vein thrombosis.

Blood transfusion is linked to the development of PGD, as well as AKI and other complications^74^, warranting a conservative strategy targeting a hemoglobin level of 7 gm/dl in the absence of cardiovascular disease and 8 gm/dl when cardiovascular disease is present^75^. It is important to recall that a reduction in blood O_2_ content due to decreased hemoglobin concentration is readily compensated by an increase in cardiac output and tissue O_2_ extraction ratio, resulting in no net reduction in oxygen consumption. Hence, the benefits of increasing O_2_ content with blood transfusion are quite limited^76^.

**Figure 3.**
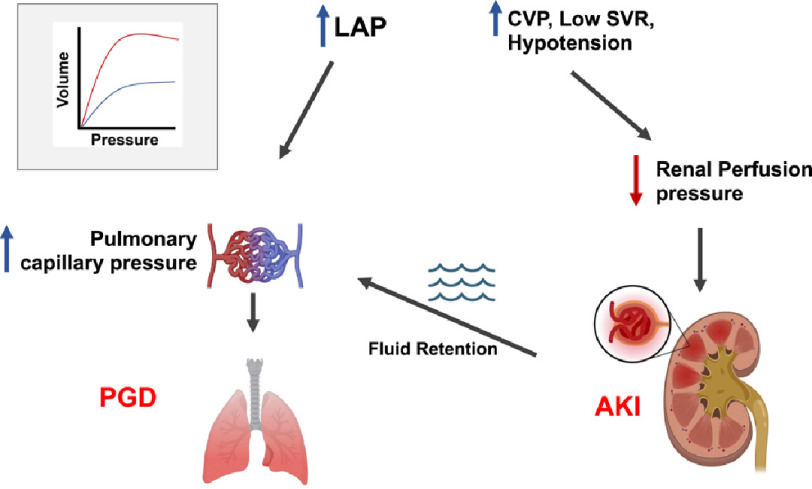
Schematic of cardiopulmonary-renal interactions early after lung transplant. Even small increases in left atrial pressure (LAP) promote primary graft dysfunction (PGD) in the setting of increased capillary permeability. Elevated central venous pressure (CVP) combined with decreased systemic vascular resistance (SVR) and hypotension results in reduced renal perfusion pressure, a key determinant of acute kidney injury (AKI). The latter leads to fluid retention, further exacerbating PGD. Upper left panel: Starling ventricular function curves: beyond a certain end-diastolic pressure or volume (pre-load) on the horizontal axis, no further increase in stroke volume is achieved. See text for further discussion. Illustration courtesy of Alexander Girgis, BS.

Ensuring an adequate preload is the first consideration in the setting of hypotension and/or oliguria. The challenge is to identify the left ventricular filling pressure that yields the optimal end-diastolic and hence the stroke volume. Exceeding this value will not further increase stroke volume, but may promote hydrostatic pulmonary edema and systemic venous congestion (Figure 3). Observations from TEE and concomitant filling pressures in the operating room can be useful in guiding early postoperative management. The use of functional hemodynamic assessments of fluid responsiveness (i.e., reduced pre-load) leads to a smaller positive fluid balance, reduced requirement for renal replacement therapy, and better outcomes in medical and surgical critical illness^77,78^.

Impaired intrinsic contractility of the right and/or left ventricle as the basis for hypotension is rare. RV function recovers rapidly once pulmonary hypertension is relieved, although transient, generally mild LV systolic dysfunction can be observed in severe PAH^50^. A slightly lower than normal cardiac index is desirable during the immediate postoperative period to avoid graft overperfusion and the potential for PGD^7^.

Reduced systemic vascular resistance is the primary mechanism for persistent hypotension after correction of hypovolemia early post-lung transplant^79^. Beyond the effects of anesthesia and sedatives^80^, a short-lived distributive or vasoplegic shock picture can be observed, occasionally profound, mediated by a systemic inflammatory response likely related to surgical trauma and/or extracorporeal circuit use^79,81^. Nor-epinephrine (NE) is the first-line vasopressor as an alpha-and potent *β*_1_-adrenergic agonist, and is therefore effective in distributive and cardiogenic shock^82^. Once the NE dose exceeds 0.05 µg/kg/min, we supplement with vasopressin, which does not raise pulmonary vascular resistance and is associated with a reduced risk of atrial fibrillation^83^. For refractory hypotension, angiotensin II is introduced, which often leads to a prompt elevation in blood pressure^84^. Avoiding high-dose catecholamines reduces their negative effects on renal perfusion through vasoconstriction of the afferent glomerular arteriole, whereas vasopressin and angiotensin II preferentially constrict the efferent arteriole, thereby potentially reducing the incidence and severity of acute kidney injury^85,86^. If additional inotropy is warranted, slow titration of dobutamine should be considered^82^. We avoid milrinone due to its potent systemic vasodilatory effects, long half-life, and lack of clinical evidence to support its use in the absence of left ventricular systolic dysfunction. Persistent RV dysfunction due to residual elevation of pulmonary vascular resistance is best managed using inhaled vasodilators. There is no evidence that epinephrine has benefits over nor-epinephrine while inducing more tachycardia, lactic acidosis^87^, and greater short-term mortality in nonsurgical cardiogenic shock^88^. Lungs physiologically produce lactate^89^ and values are higher at the end of surgery in subjects who develop severe PGD^90^, reducing the specificity of this biomarker for tissue hypoperfusion. Values should be taken in the context of other indices of tissue perfusion. Prolonged peri-operative shock is often accompanied by variable degrees of hepatic impairment which further complicates critical illness and management^91^.

*Atrial arrhythmia*

Atrial fibrillation (AF) and/or flutter are common, with an incidence of approximately 30% in the early post-lung transplantation period. Risk factors include age and underlying heart disease. AF has a significant impact on peri-operative mortality (odds ratio: 2.7) and length of stay^92^. Prevention of AF in patients at increased risk is warranted^93^. Beta-blockers should be continued postoperatively for patients undergoing chronic therapy. Persistent sinus tachycardia is a common antecedent of AF during the early postoperative period. Once volume status and pain control have been optimized, a low threshold to introduce beta-blockers should be maintained, as lung transplantation (particularly bilateral) is often associated with some degree of vagal denervation. A lower incidence of AF was observed in a randomized trial of vasopressin vs. norepinephrine for vasoplegic shock after cardiac surgery^94^. The use of dexmedetomidine for sedation may be associated with less AF after cardiac surgery than propofol^95^.

A strategy of rate vs. rhythm control results in earlier restoration of sinus rhythm after cardiac surgery, but no difference in clinical outcomes or proportion without AF at hospital discharge^96^. In our experience, rate control often fails and rapid AF is poorly tolerated in these patients. Relapse after spontaneous resolution is common. Successful early anti-arrhythmic therapy with or without direct-current cardioversion results in better hemodynamic stability and eliminates the need for anticoagulation and the attendant risk of surgical bleeding if the duration of AF is <48 h^96^. When AF is persistent or recurrent, we carefully balance the risk of systemic embolism versus surgical bleeding and generally avoid anticoagulation, as spontaneous hemothorax is a major threat in the early postoperative period. In recipients with a history of AF, we perform AtriClip left atrial appendage exclusion if feasible. Recent data suggest that the risk of thromboembolism is comparable to that of anticoagulation in AF after cardiac surgery^97^. Some reports have suggested a link between amiodarone use and increased mortality within 1 year^98–100^; however, it is likely that the population in whom amiodarone use was deemed necessary had other confounders. This association has not been uniform^101^, and in our experience, the short-term (30 days) use of amiodarone has been well tolerated with good intermediate-term outcomes.

### Acute kidney injury (AKI)

AKI (Table 6) is exceedingly common after LTX and has a significant impact on both short- and long-term outcomes. In a large meta-analysis, the incidence of AKI was 53% (95% CI [46–59]%), and renal replacement therapy (RRT) use ranged from 4–15%^102^. The pooled odds ratios for 1-yr mortality were 2.99 for any AKI and 8.32 for RRT. However, a causal link between AKI and early mortality is not always evident. Renal dysfunction, despite RRT, is associated with fluid overload and metabolic derangements that can promote other complications such as pulmonary edema, infection, and cardiovascular events.

**Table 6:** Kidney disease: improving outcomes criteria for acute kidney injury^a^.

Stage	Serum creatinine	Urine output
1	≥0.3 mg/dl rise in 48 hr *or* 1.5 –1.9 × baseline within last 7d	<0.5 ml/kg/h for ≥6 h
2	2.0 –2.9 times baseline	<0.5 ml/kg/h for ≥12 h
3	≥3 times baseline or ≥4.0 mg/dl or initiation of renal replacement therapy	<0.3 ml/kg/h for ≥24 h or anuria for ≥12 h

**Notes.**

a Onset within 7 days of transplant surgery.

PGD and AKI often occur concomitantly in critically ill patients after complicated lung transplant procedures. In a single-center analysis, postoperative AKI developed in 63% of the LTX recipients. Severe (grade 3) PGD was observed in 27% of patients with AKI vs. 9% of those without AKI^103^. Moreover, the likelihood of severe PGD increased with greater severity of renal impairment, occurring in two-thirds of patients with stage 3 AKI. PGD and AKI were independently associated with one-year survival and the highest mortality rate was observed among recipients with both complications. However, the basis for this association remains unclear. Both may be the result of endothelial injury and systemic inflammation, which share several underlying risk factors. Renal dysfunction and oliguria may prompt fluid loading and promote alveolar edema formation in the presence of increased pulmonary capillary permeability. PGD is often associated with hemodynamic derangements and the need for higher-intensity mechanical ventilation, which could result in renal hypoperfusion.

Beyond its short-term impact, the link between postoperative AKI and its adverse long-term consequences is likely underappreciated*.* Chronic kidney disease (CKD) is highly prevalent in LTX recipients. In a large single-center study, any degree of postoperative AKI was a strong independent predictor of subsequent CKD stages 4–5 (estimated glomerular filtration rate [eGFR] < 30 ml/min/1.73 m^2^), as well as long-term mortality, even after excluding those who required postoperative RRT, developed end-stage renal disease, or died during the initial hospital stay^104^. A population-based cohort analysis of non-renal organ transplant recipients from the 1990’s found a relative risk of death after development of advanced CKD stages of 4.55^105^. Similar to AKI, mortality associated with CKD is often not directly ascribed to renal dysfunction. CKD typically leads to a reduction in calcineurin inhibitor target concentrations, which could promote chronic lung allograft dysfunction (CLAD), the main cause of long-term mortality. Multiple complications are associated with CKD, including a high incidence of serious cardiovascular events^106^.

The pathogenesis of postoperative AKI generally involves renal hypoperfusion, which leads to altered renal function and structure^107^. Additional factors include systemic inflammation and toxin-induced renal injury. Several pretransplant recipient-related risk factors have been identified with variable strengths of association. These include reduced eGFR, hypertension, diabetes, age, non-Caucasian race, body mass index (BMI), pulmonary hypertension, and need for ICU care and ECMO support. Despite their importance, procedure-related variables have not been studied adequately. Limited data suggests that intra-operative hypotension^108^, vasopressor use^109^, duration of surgery and intra-operative hypoxemia^110^ are risk factors. The use and duration of extracorporeal life support (ECLS, CPB, or ECMO) are likely to be relevant. Some studies have suggested an increased incidence with bilateral vs. single lung transplants^102^, perhaps related to the increased use of ECLS in the former. Supratherapeutic CNI concentrations have been associated with AKI^111^, but other early postoperative events and management, such as hemodynamics, fluid balance, and anti-microbials, have not been well characterized.

Considerable progress has been made in recent years in cardiac surgery-associated AKI (CSA-AKI), which shares many similarities with LTX^112,113^, such as hemodynamic instability, use of cardiopulmonary bypass, and pre-existing co-morbidity. CPB is associated with circulatory alterations, in part because of the loss of pulsatile flow^114^. Contact activation in the CPB or ECMO circuit induces the release of inflammatory mediators, complement activation, and hemolysis, all of which can contribute to renal injury. Pre-operative anemia and peri-operative blood transfusions are both linked with CSA-AKI^115,116^. Transfusion during ECLS use may be particularly injurious, as stored red blood cells are more susceptible to hemolysis, leading to scavenging of nitric oxide by free hemoglobin and other deleterious effects that could contribute to AKI^117^. Recognition of these and other clinical determinants has led to the development of validated risk scores and institution of care bundles that have resulted in improved outcomes in CSA-AKI^112,118^. A commercially available biomarker of tubular epithelial cell damage (the product of tissue inhibitor of metalloproteinase-2 and insulin-like growth factor-binding protein 7 [TIMP-2*IGFB7] in urine) can be used to identify AKI prior to changes in serum creatinine levels or urine output. Rapid institution of renoprotective strategies based on biomarker results has been shown to reduce the incidence and severity of CSA-AKI^118,119^; however, this approach remains to be validated post-LTX.

The Kidney Disease: Improving Global Outcomes (KDIGO) group has created extensive guidelines for the prevention and management of AKI in high-risk critical illnesses, which can be applied to all LTX recipients^120^. General principles include 1) optimization of volume status and renal perfusion, 2) avoidance of nephrotoxic agents when possible, and consideration of alternatives to radiocontrast procedures, and 3) glycemic control. Close monitoring of serum creatinine levels, urine output, and hemodynamics is essential. Functional hemodynamic monitoring may be useful to ensure adequate preload while avoiding venous congestion and consequent reduction in renal perfusion pressure (Figure 3). Fluid accumulation is associated with the progression of AKI and worse outcomes in critical illness^121,122^. CVP > 10 mmHg has been associated with a higher incidence of AKI and mortality following cardiac surgery^123^ and LTX recipients with CVP > 7 mmHg had higher peak serum creatinine levels in the intensive care unit^60^. The potential impact of various vasopressors and inotropes should be considered. Vasopressin and angiotensin II have favorable effects on renal perfusion pressure, whereas alpha-adrenergic agonists may be deleterious^85^. Milrinone improves renal blood flow and decreases renal vascular resistance in LV systolic dysfunction. However, a decrease in arterial pressure would offset any benefits to renal function and may require concomitant administration of nor-epinephrine to maintain renal perfusion pressure^124^.

Many centers delay the introduction of a calcineurin inhibitor in the setting of hemodynamic instability or AKI, which is feasible when an induction agent, such as basiliximab, has been administered^125^. The addition of co-stimulation blockade with belatacept allows prolonged reduction of calcineurin inhibitor exposure, facilitating the recovery of renal function while providing adequate immunosuppression^126,127^. However, an increased risk of rejection and EBV-associated post-transplant lymphoproliferative disorder has been noted with this strategy^127,128^ and the long-term use of belatacept may be associated with increased mortality in LTX recipients^129^. Vancomycin has the potential for nephrotoxicity^130^, particularly with concomitant piperacillin-tazobactam^131^ and this combination should be avoided if possible. Extreme caution should also be exercised when considering other nephrotoxic anti-microbials, such as aminoglycosides and amphotericin B^132^.

## Summary

PGD and AKI are extremely common and intertwined conditions during the early post-lung transplant period and often lead to prolonged ICU stay, additional complications and markedly increase the risk of hospital mortality. Long-term morbidity and mortality rates are also affected. Both are driven, in part, by circulatory derangements. Meticulous monitoring and optimization of respiratory status, hemodynamics, and renal function are critical to prevent and attenuate the severity of these complications and to improve both short- and long-term outcomes after lung transplantation.
